# Society of Biology

**Published:** 1879-05

**Authors:** O. C. Oliver

**Affiliations:** Hotel D’Orient Paris


### ARTICLE XI.

SOCIETY OF BIOLOGY. February 2, 1879. President, M. Paul Bert.

*Contractions due to Intra-ventricular Hæmorrhages.*—M. Duret, à propos of a communication made by M. Cassey at a previous meeting, said he had made experiments upon animals which seemed to him to disprove the theory of contraction advocated by M. Cassey. The speaker had found an injection of either water, wax or oil, provided it was made rapidly, produce an immediate and intense tetanism, with considerable elevation of the arterial tension. He attributed this contraction to reflex irritation of the corpora restiformia, or the sensitive portions of the meso-cephalon. Mechanical excitation, executed experiment ally, of the corpora restiformia, sufficed to produce a pronounced episthotonos. Hence the speaker concluded that in intra-ventricular hæmorrhage, the contraction is the result of the reflux of the sub-arachnoid fluid and shock of the corpora restiformia, or to the direct excitation of the blood which, by the aqueduct of Sylvius, flows into the fourth ventricle. M. Duret had undertaken these experiments to verify his theory of cephalo-rachidian shock. He has by intra-ventricular injections, simulated the displacement of the sub-arachnoidean fluid, and in the two cases the phenomena were the same, to wit, violent tetanism.

M. Rochefontaine could not admit the theory of M. Duret, as, according to the speaker's observation, there was very often no fluid in the cerebral ventricles of the dog; any one could convince himself of this by observation. M. Duret replied that in the ventricular cavities and around the medulla oblongata, there existed in the cadaver, empty spaces, yet it should not be inferred from this that they existed in the living animal. The elementary laws of physics and physiology required that these cavities should be filled by a liquid. To establish the presence of a sub-arachnoidean fluid, M. Rochefontaine had placed the subject under very unfavorable experimental conditions; in thus opening freely the cranium, and anæsthetizing the animal, he changed the intra-cranial arterial tension, consequently modifying the quantity, normally existing, of the sub-arachnoidean fluid. By reason of the opening into the cranium, an enormous turgescence of the brain was induced, displacing the fluid normally existing in the ventricles, and driving it into the sub-arachnoidean space. M. Duret proposed to further investigate this interesting and difficult question, and present his results at a subsequent meeting of the society.

*Hygiene of Sight.*—M. Javal made a statistical report upon certain alterations of vision in children, embracing a very large number of cases. In every case in which the vision is not absolutely good, an examination revealed astigmatism in the children, by correcting which, normal vision was established. At the school Mouge, where the lighting is excellent, M. Javal has found ten per cent. of the children astigmatic, but thinks it probable that they had become so previous to entering the school. The speaker then gave the reason why books printed on yellowish paper were less fatiguing to the eyes. Though the astigmatism was corrected, there was the error of chromatism which they had scarcely dreamed of combating. Yet a little reflection showed that this might be corrected or reduced to a minimum by the employment of mono-chromatic light, but as it was desirable to suppress the least possible amount of light consistent with the correction, it was advisable to suppress one or the other extremity of the spectrum. If the red end be eliminated, too much light is lost. The desired object is much more satisfactorily attained by absorption of the blue and violet rays, the resulting tint being a yellowish grey. Such is the true reason of the superiority of paper tinted as above for readers' and students' use.

*Fatty Emboli in Fractures and Osseous Degenerations.*—M. Déjerine said he had published the notes of two cases in November last, in which he had observed fatty emboli following osseous degenerations. Since that time he has had occasion to observe six others. In examining the lungs, he has each time very clearly established the existence of fatty emboli, proportioned in quantity to the extent and intensity of the osseous degeneration. He has also found, as stated by the authors, that the emboli were less marked the longer the time which had elapsed between the traumatism and death. In two cases only, has he found fatty emboli in the liver and kidneys. He has not had occasion to observe a case in which it was general, as has been many times seen in Germany. M. Déjerine, moreover has made experiments upon fatty embolism in the laboratory of M. Vulpian, using dogs as subjects of his experiments. In varying the operative procedure, he has been able to produce fatty embolism in varying degrees in these animals, from the slightest to the most severe forms, such as observed in man after large wounds of the bones. The mode of procedure followed by the observer, consisted in production of simple fractures, without communication with the exterior, in which cases the fatty emboli of the lungs were very slight, and in some cases hardly perceptible. When, on the contrary, the marrow was implicated, in whole or in part, by the introduction of a foreign body into the medullary canal, the fatty embolism was very marked, and the fatty particles could be traced in the blood from the veins of the wounded member to the vessels of the lungs. If, instead of introducing an inert body such as a piece of iron, a substance such as a piece of laminaria digitalis be inserted, the embolism was exceedingly violent, the lungs being literally injected with fat. These observations confirm those of Bergmann and Halm. M. Déjerine remarked that the embolism produced by the introduction of pieces of laminaria into the medullary canal of the bone, was much more pronounced than that produced by other experimental procedures. It appeared to the speaker that the fatty embolism in man was probably successive to a rapidly developed osteo-myelitis, which by pressure from within and without, forced the fat into the osseous capillaries, and from there into the venous circulation. In the animals experimented upon, it is difficult to produce a veritable osteo-myelitis and we should not demand more from experimental pathology than it is able to give; but by introducing in the entire length of the medullary canal a piece of laminaria we produce a persistent irritation of the medulla, as well as an eccentric compression of the medullary canal. These experiments seemed to the speaker to confirm the theory already enunciated, of the pathology of fatty embolism, as in the experiments with the laminaria one of the conditions, at least, was paramount, namely, that of tension, and as fatty embolism succeeded this tension, it was fair to infer that this was one of the essential elements in its production.

O. C. OLIVER.

HOTEL D'ORIENT PARIS, March 5, 1879.

